# Increase in Chymase-Positive Mast Cells in Recurrent Pleomorphic Adenoma and Carcinoma Ex Pleomorphic Adenoma of the Parotid Gland

**DOI:** 10.3390/ijms222312613

**Published:** 2021-11-23

**Authors:** Ichita Kinoshita, Denan Jin, Masaaki Higashino, Tetsuya Terada, Yoshitaka Kurisu, Shinji Takai, Ryo Kawata

**Affiliations:** 1 Department of Otorhinolaryngology-Head and Neck Surgery, Osaka Medical and Pharmaceutical University, Takatsuki-City 569-8686, Japan; ichita.kinoshita@ompu.ac.jp (I.K.); masaaki.higashino@ompu.ac.jp (M.H.); tetsuya.terada@ompu.ac.jp (T.T.); ryo.kawata@ompu.ac.jp (R.K.); 2 Department of Innovative Medicine, Graduate School of Medicine, Osaka Medical and Pharmaceutical University, Takatsuki-City 569-8686, Japan; shinji.takai@ompu.ac.jp; 3 Department of Pathology, Osaka Medical and Pharmaceutical University, Takatsuki-City 569-8686, Japan; yoshitaka.kurisu@ompu.ac.jp

**Keywords:** mast cell, chymase, pleomorphic adenoma, carcinoma ex pleomorphic adenoma, parotid tumor

## Abstract

Incomplete excision of pleomorphic adenoma (PA) may result in recurrent pleomorphic adenoma (RPA). Furthermore, long-term neglected PA may become carcinoma ex pleomorphic adenoma (CXPA). In the present study, the relationships between mast cell-derived chymase and these tumors were examined. The tumor tissues of PA consisted of either or both glandular and fibrotic structures. Histological features of RPA were almost similar to those of PA, except that they showed multinodular structures. CXPA is composed of a mixture of PA and carcinoma. The main stromal cells in PA were myofibroblasts, whereas fibroblasts constituted the main cellular portion in the stromal tissue of RPA. Cancer-associated fibroblasts (CAFs) were present abundantly in CXPA. With increased VEGF expression, neovascularization tended to increase in RPA or CXPA. Compared with PA, chymase-positive mast cells, as well as chymase gene expression, were increased in the tumor tissues from patients with RPA or CXPA. SCF, TGFβ1, and PCNA-positive staining was widely observed in these tumor tissues. The above results suggest that mast cell-derived chymase through its direct or cooperative effects with other mediators may participate in the pathophysiology of RPA and CXPA.

## 1. Introduction

Pleomorphic adenoma (PA) is the most common histological type of parotid tumor, and most cases can be completely cured by surgical removal. However, despite them being benign tumors, recurrence may occur in a few cases (RPA). The frequency of recurrence is said to be 2% to 8% [1,2,3]. As surgical factors, capsule damage and enucleation are considered [2]. On the other hand, tumor factors include tumor size, histopathological subtype, satellite nodule, and deep lobe tumor. In addition, RPA is presumed to have higher tumor activity, but its biological mechanism is unknown [4]. In addition, it is known that PA can become carcinoma ex pleomorphic adenoma (CXPA) in the long term. It is reported that 9.6% of PAs become CXPAs if PAs are left untreated for 15 years or more [5]. As is well known, malignant transformation is more frequent in RPA [3]. The cause is considered to be not only that it is left for a long time, but also high tumor activity [6,7]. However, not all recurrent cases become malignant, and it seems that there is a different biological mechanism in malignant transformation.

Recently, research on the relationship between mast cells and tumor pathology has attracted a great deal of attention. Mast cells contain preformed mediators, such as histamine, tryptase, and chymase, and they can also release some de novo synthesized mediators, such as tumor necrosis factor (TNF) α and transforming growth factor (TGF)-β1 [8]. Some of these mediators seem to be important in the development of the tumor microenvironment (TME) [9]. Within the tumor, mast cell interactions occur with infiltrated immune cells, tumor cells, and extracellular matrix (ECM) through direct cell-to-cell interactions or release of a broad range of mediators capable of remodeling the TME [9]. Of these mast cell-derived mediators, chymase has various actions and has attracted our interest for a long time. For example, chymase can, through the activation of stem cell factor (SCF), regulate mast cell numbers [10], and, as is well known, the numbers of mast cells have been reported to be increased in several kinds of human cancers, such as malignant melanoma, breast carcinoma, and colorectal adenocarcinoma [11,12]. Moreover, chymase, through its enzymatic activity, can cleave angiotensin (Ang) I to Ang II, as well as latent TGF-β1 to its active form [13,14]; these mediators are believed to be important in angiogenesis. On the other hand, chymase can also cleave pro-matrix metalloproteinase (MMP)-9 to MMP-9 [15], which is well known as a potential biomarker of cancer invasion and metastasis. We have found that chymase-positive cells were significantly increased in human lung and gastric cancers [16,17], indicating the importance of chymase in such conditions. However, up to now, few studies have evaluated the distribution characteristics of mast cell-derived chymase in parotid tumors.

RPA and CXPA are less common, and the long-term accumulation of cases is needed for their examination. This time, it was possible to accumulate enough cases for evaluation. In this study, it was thought that comparative investigation of chymase and related substances in PA, RPA, and CXPA could elucidate one of the biological mechanisms of tumor activity in RPA and CXPA. Therefore, the distribution pattern and expression of chymase were examined in fresh PA, RPA, and CXPA cases, and they were compared with their clinical features.

## 2. Results

### 2.1. Subject Enrolment

Specimens from a total of 60 patients (29 males, 31 females) were included in the present study. As shown in Table 1, there were no significant differences in the sex distributions found between the PA and RPA or CXPA groups. No significant differences in the age distribution were also found between the PA and RPA or CXPA groups. In the CXPA group, 10 cases showed invasive malignant tissues beyond the primary tumor. In the remaining seven cases, there were no invasive malignant tissues involving the surrounding normal tissues. The histopathological types of the malignant component of CXAP included four cases of myoepithelial carcinoma, six cases of adenocarcinoma, NOS, five cases of salivary duct carcinoma, and two cases of others.

### 2.2. Histological Features of PA, RPA, and CXPA

**Figure 1:**
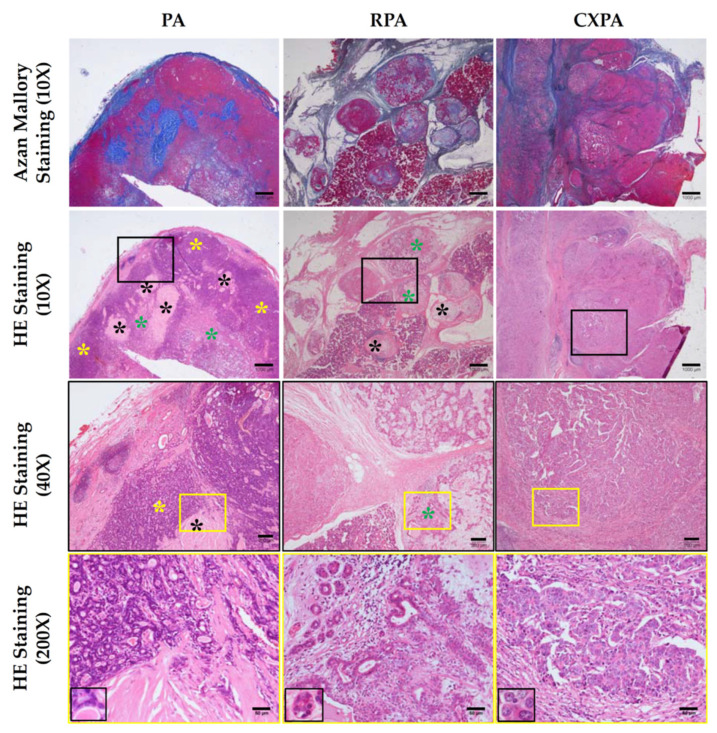
Representative Azan Mallory-stained and HE-stained cross-sections from patients with PA, RPA, or CXPA. Yellow asterisks indicate the glandular structures in PA. Black asterisks indicate fibrotic structures in PA and RPA. Green asterisks indicate a mixture of fibrotic and glandular structures in PA and RPA. The scale bars in the magnifications of 10×, 40× and 200× indicate 1000 μm, 200 μm and 50 μm, respectively.

Figure 1 shows the representative Azan Mallory-stained and HE-stained cross-sections from patients with PA, RPA, or CXPA. As can be seen in the Azan Mallory staining of Figure 1, the tumor tissues of PA were wrapped by a thick connective tissue-like capsule, while no series of connective tissues could be found in the circumferences of invasive CXPA. The tumor tissues of PA consisted of either glandular or fibrotic structures, as well as their mixed tissue structures (HE staining of Figure 1). As can be seen in the high-power field (200×) of HE staining in Figure 1, the trace of mucoid retention was surrounded by the secretions of epithelial cells in the glandular structures. RPA showed multinodular structures, and the tumor tissues were segmentally covered with fibrotic capsules (Azan Mallory staining of Figure 1). As shown in the photograph, the aniline blue color for collagen deposition is relatively light. The tissue constituents of RPA were similar to those of PA (HE staining of Figure 1). CXPA, by definition, is composed of a mixture of PA and carcinoma on microscopic examination. The photograph of CXPA shown in Figure 1 is mainly focused on the carcinoma region, and upon pathological examination, the main malignant component of CXPA was diagnosed as salivary duct carcinoma. As can be seen in the HE staining, the carcinoma grew to occupy the entire neoplasm, leaving no trace of the PA component. Among these regions, moderate blue staining was mixed in tumor parenchyma tissues (Azan Mallory staining of Figure 1), which may indicate that they were scar tissues that were replaced after necrosis of the carcinoma tissues or residual fibrotic tissues from PA.

### 2.3. Identification of Fibroblasts and Cancer-Associated Fibroblasts

**Figure 2:**
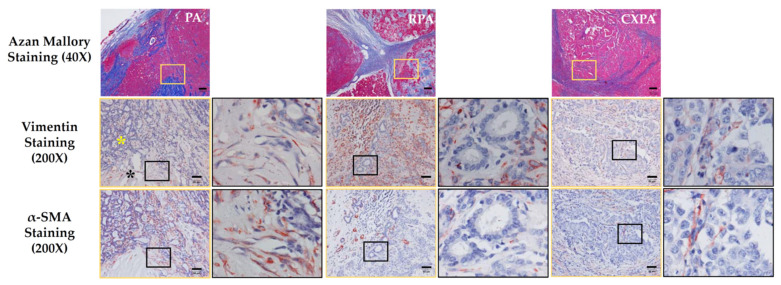
Representative vimentin and α-SMA immunostaining of serial cross-sections from patients with PA, RPA, or CXPA. These loci were matched to the yellow frames of Azan Mallory-stained sections shown in the upper column. The immunostained images surrounded by yellow frames are 200× magnification, and black frames are 1000× magnification. Yellow asterisks indicate the glandular structures in PA. Black asterisks indicate fibrotic structures in PA. The scale bars in the magnifications of 40× and 200× indicate 200 μm and 50 μm, respectively.

Figure 2 shows representative vimentin and α-SMA-immunostained serial cross-sections from patients with PA, RPA, or CXPA. The loci were matched to the yellow frames of Azan Mallory-stained sections, as shown in the upper column of Figure 2. Vimentin is a marker protein for mesenchymal origin cells that are mainly expressed in fibroblasts and myofibroblasts, as well as endothelial cells [18]. While α-SMA as a contractile protein is mainly expressed in contractile vascular smooth muscle cells, it is also expressed in myofibroblasts after the phenotypic change from fibroblasts has occurred [19]. Therefore, the proportions of fibroblasts and myofibroblasts among tumors can be calculated with the combination of vimentin and α-SMA immunostaining. As shown in Figure 2, the vimentin-positive cells in the PA tissues were scattered diffusely in both glandular (yellow asterisks) and fibrotic structures (black asterisks). In addition, α-SMA immunostaining performed in the serial section adjacent to the vimentin immunostaining confirmed that most of these α-SMA-positive cells overlapped with the vimentin-positive cells, indicating that most of these stromal cells were myofibroblasts. On the other hand, the vimentin-positive cells in the RPA tissues were stained more densely than other tumors, and as shown in the enlarged HPF of the lower photographs, the vimentin-positive cells were mostly negative for α-SMA, indicating that most of these stromal cells were fibroblasts. The vimentin-positive cells in the CXPA tissues were moderately scattered among cancer cells, as well as their surrounding fibrotic regions, and some of them were also positively stained with α-SMA, indicating that cancer-associated fibroblasts (CAFs) were present abundantly in the malignant parts of CXPA. The concept of CAFs is new, and the amount of this population was reportedly correlated with a poor prognosis in malignancy [20].

### 2.4. Examination of Neovascularization and Proliferative Cells

**Figure 3:**
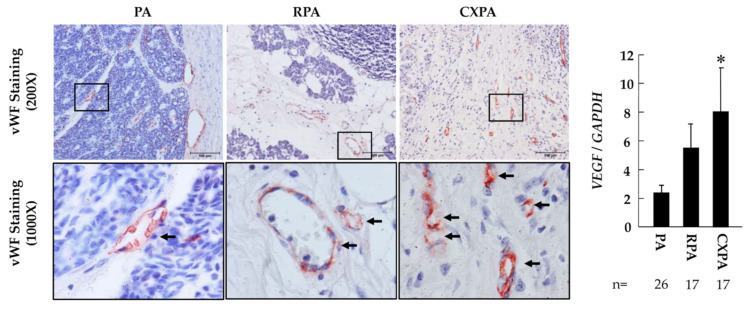
Representative vWF immunostaining, as well as gene expression levels of *VEGF*, in the tumor tissues from patients with PA, RPA, or CXPA. Black arrows represent microvessels. * *p* < 0.05, vs. PA. The scale bars in the magnification of 200× indicate 100 μm.

Figure 3 shows representative vWF immunostainings, as well as the gene expression levels of *VEGF*, in the tumor tissues from patients with PA, RPA, or CXPA. VEGF is a product of macrophages and tumor cells and acts as a very potent angiogenic agent under the malignant tumor condition [21]. As can be seen in the bar graph of Figure 3, compared to PA, the *VEGF* expressions in RPA or CXPA tended to increase or be increased significantly in RPA and CXPA. In addition, vWF is a marker of vascular endothelial cells, and it is routinely used to identify vessels in tumor tissues [22]. As can be seen in the vWF immunostainings, compared to PA, denser small microvessels were found in the tumor tissue from the cross-sections of patients with CXPA and RPA.

**Figure 4:**
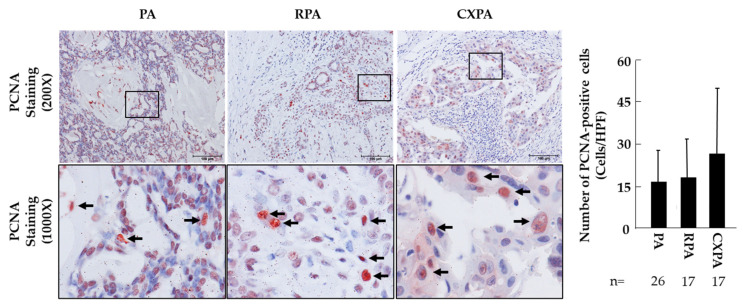
Representative PCNA immunostaining, as well as the estimated numbers of PCNA-positive cells, in the tumor tissues from patients with PA, RPA, or CXPA. Black arrows indicate positive for PCNA antigens. The scale bars in the magnification of 200× indicate 100 μm.

Figure 4 shows representative PCNA immunostainings, as well as the estimated numbers of PCNA-positive cells, in the tumor tissues from patients with PA, RPA, or CXPA. PCNA is a key component of the DNA replication machinery involved in the process of DNA elongation, recombination, methylation, and repair. PCNA expression is high in proliferating cells during the G1 and S phases of the cell cycle [23]. Therefore, the increase in PCNA-positive cells means that the cells are in a proliferative cycle. As can be seen in Figure 4, the PCNA-positive cells could be found in all cross-sections from patients with PA, RPA, or CXPA, and quantitative analysis confirmed that there were no significant differences among the three groups examined (bar graph of Figure 4). However, the type of PCNA-expressing cells was quite different. In PA and RPA, PCNA-positive staining was mainly localized in the epithelial cells of glandular structures, as well as in the fibroblast-like spindle cells of fibrotic structures, whereas in CXPA, PCNA-positive staining was mainly localized in cancer cells of malignant structures and to some extent in CAF-like cells.

### 2.5. Identification of Fibroblasts and Cancer-Associated Fibroblasts

**Figure 5:**
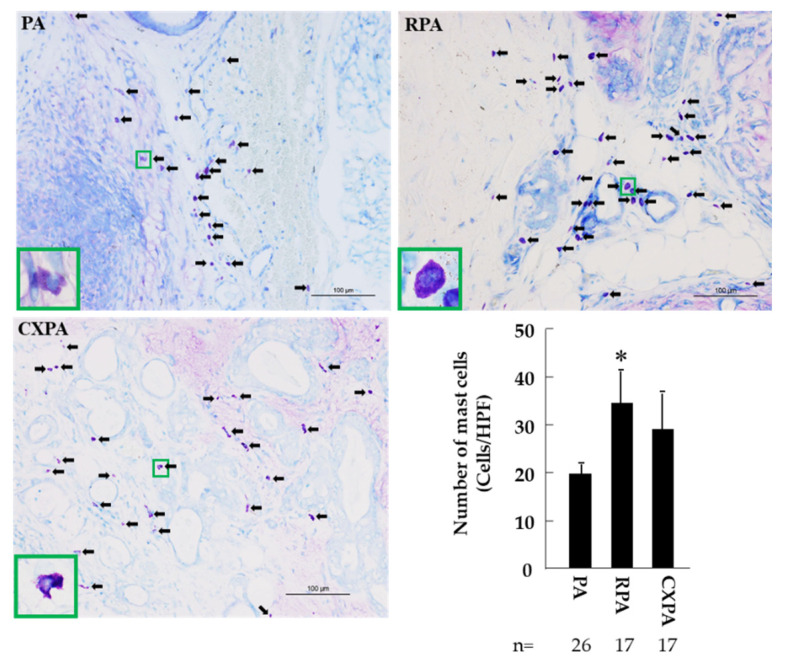
Representative toluidine blue staining, as well as the calculated number of mast cells, in the tumor tissues from patients with PA, RPA, or CXPA. Black arrows indicate mast cells. As can be seen in these photographs, the cytoplasm of mast cells is stained purple with toluidine blue. * *p* < 0.05 vs. PA.

**Figure 6:**
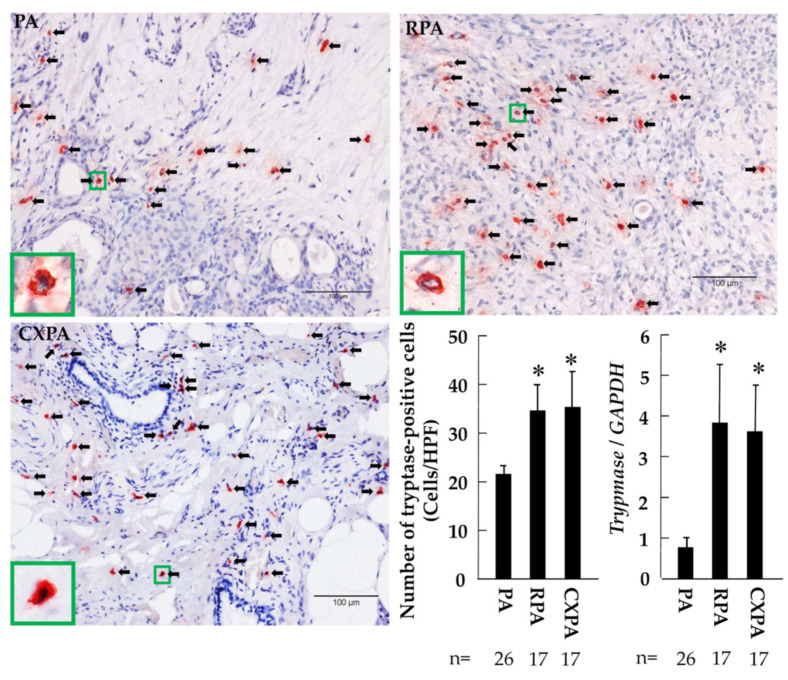
Representative tryptase immunostaining and the calculated number of tryptase-positive cells, as well as *tryptase* gene expression, in the tumor tissues of patients with PA, RPA, or CXPA. Black arrows indicate the tryptase-positive cells. * *p* < 0.05 vs. PA.

**Figure 7:**
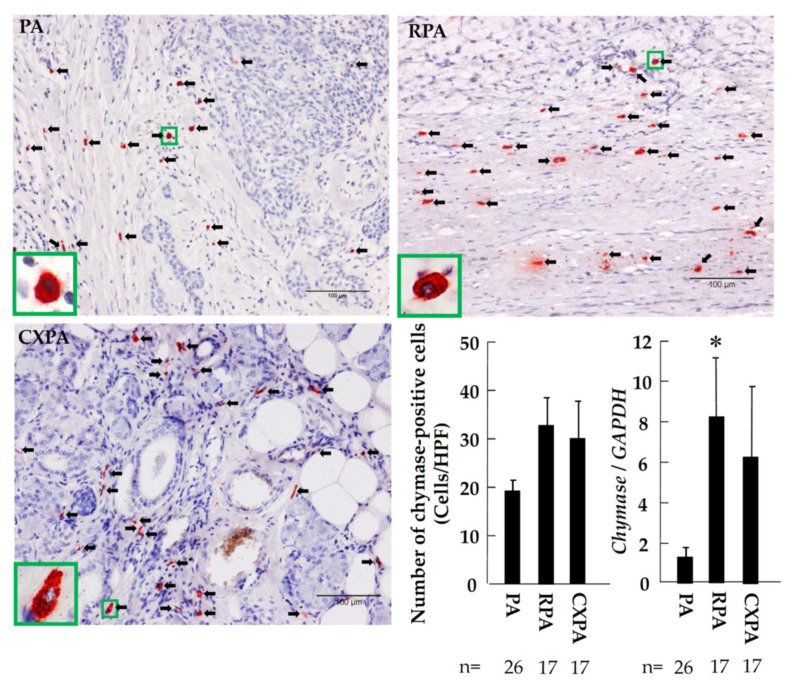
Representative chymase immunostaining and the calculated number of chymase-positive cells, as well as *chymase* gene expression, in the tumor tissues from patients with PA, RPA, or CXPA. Black arrows indicate the tryptase-positive cells. * *p* < 0.05 vs. PA.

**Figure 8:**
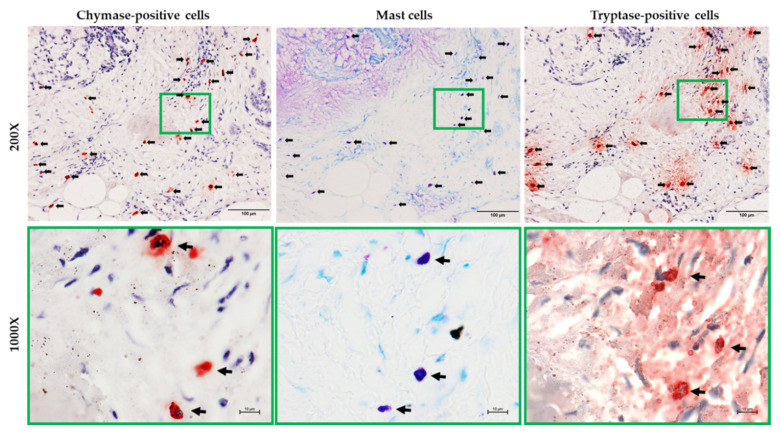
Representative toluidine blue, chymase, and tryptase-stained serial cross-sections positioned before and after the toluidine blue-stained sections. Black arrows indicate tryptase-positive cells. Mast cells confirmed by toluidine blue staining are almost in the same position as the chymase- and tryptase-positive cells (HPF 1000×), indicating that mast cells are the main cellular source in these tumors. The scale bars in the magnifications of 200× and 1000× indicate 100 μm and 10 μm, respectively.

Figure 5 shows representative toluidine blue staining, as well as the calculated number of mast cells, in the tumor tissues from patients with PA, RPA, or CXPA. As can be seen in these photographs, the cytoplasm of mast cells was stained purple with toluidine blue, which makes it easy to distinguish the mast cells from the others. As shown in the bar graph of Figure 5, the numbers of mast cells in the PRA and CXPA groups were significantly increased or tended to increase compared to in the benign tumor of PA. Similar to the number of mast cells calculated in the three groups examined, the numbers of tryptase-positive cells in the RPA and CXPA groups were also significantly higher than in the PA group (Figure 6). Compared with the PA group, a significant increase in *tryptase* gene expression in the tumor tissues of RPA and CXPA was also observed (Figure 6). Figure 7 shows the chymase immunostaining and the calculated number of chymase-positive cells, as well as the gene expression of *chymase*, in the tumor tissues from patients with PA, RPA, or CXPA. As shown in the bar graph, the number of chymase-positive cells and the gene expression level of *chymase* tended to be or were significantly increased in the RPA and CXPA groups compared to the PA group. As is well known, mast cells may contain both tryptase and chymase in their secretory granules (MC_TC_ cells) or tryptase, but not chymase (MC_T_ cells) [24]. To clarify which types of mast cells were expressed in the respective tumor tissues, chymase and tryptase immunostaining of the serial cross-sections positioned before and after the toluidine blue staining were performed. Since the dimeter of mast cells is about 20 μm, and the cross-sections were about 4 μm thick, the rate of appearance of the same mast cell in three respective serial cross-sections is comparatively high. As can be seen in Figure 8, the locus of mast cells confirmed by toluidine blue staining was very similar to that of chymase- and tryptase-positive cells (HPF 1000×), indicating that mast cells were the main cellular source in these tumors. Since no significant differences could be found in the mean numbers of mast cells and chymase- and tryptase-positive cells, in the three respective tumor types, all of these mast cells that appeared in these tumor tissues were the MC_TC_ type of mast cells.

### 2.6. Identification of SCF- and TGFβ1-Positive Cells

**Figure 9:**
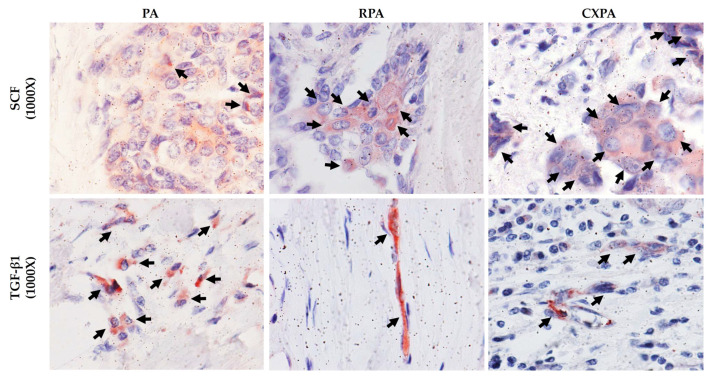
Representative SCF and TGFβ1 immunostaining in the tumor tissues from patients with PA, RPA, or CXPA. Black arrows indicate SCF- and TGFβ1-positive cells.

Figure 9 shows representative SCF and TGFβ1 immunostaining in the tumor tissues from patients with PA, RPA, or CXPA. As can be seen in these photographs, the glandular epithelial cells in PA and RPA, and the cancer cells in CXPA were mainly positively stained with SCF, whereas the fibroblast-like spindle cells were the main cellular source of TGFβ1 in all three tumor types examined (Figure 9).

## 3. Discussion

Since PA is a benign tumor, it had a good prognosis if appropriate resection is performed. However, it sometimes recurs, despite its benign nature, with a reported frequency of 2–8% [1,2,3]. Since RPAs frequently have multiple nodules [25,26], and the surgical wound is scarred, tumor removal at reoperation is not easy, and it is often difficult to preserve the facial nerve, unlike at the initial surgery [27]. Therefore, the incidence of postoperative facial nerve paralysis is higher than that following fresh PA [27,28]. It is presumed that the cause of recurrence is related not only to surgical procedure problems such as enucleation and cell dissemination [2], but also to the nature of the tumor. In fact, there are many cases in which recurrence does not occur despite enucleation, and tumor factors may also be considered as a cause of recurrence, that is, higher tumor activity. PA is also known to cause malignant transformation. This malignant transformation may occur in 1.6% of PA patients with a clinical duration less than 5 years, but it increases greatly to 9.6% when the clinical duration is over 15 years [5]. RPA generally has a long term, so the frequency of malignant transformation is high [3]. Although CXPA is thought to have higher tumor activity [29], the exact molecular mechanism involved in mediators of tumor growth progression and malignant transformation remains unclear. When PA is the basic pathological condition, RPA and CXPA can be regarded as biologically active forms of PA, and it is considered extremely useful clinically to elucidate the mechanism.

Cumulative histological clinical studies have shown an increase in mast cells in some types of malignant tumors. Evidence from many basic experiments indicates that some mediators contained in mast cells, such as TNF-α, prostaglandins, VEGF, tryptase, and chymase, seem to be important in promoting tumor hyperplasia and invasive and metastatic processes, as well as creating the TME [9]. In the present study, regardless of whether the tumors were benign or malignant, most mast cells were the MC_TC_ phenotype, and these cells were significantly increased in the tumor tissues from patients with either RPA or CXPA. As is well known, chymase is stored in the granules of MC_TC_ mast cells [24] and released with other mediators by degranulation under certain conditions. As mentioned above, chymase could cleave Ang I to Ang II very efficiently in local tissues [13], and this chymase-mediated Ang II-forming pathway was reported to be very important in promoting neovascularization. For example, Muramatsu et al. [30] have reported that the angiogenesis in an Ang I-infused sponge-implanted model was markedly suppressed by treatment with a chymase-specific inhibitor, and this suppression was achieved through the inhibitory effects on the chymase-Ang II-VEGF-dependent pathway, indicating that chymase-generated Ang II participates in the neovascularization process through the increase in VEGF expression. As is well known, both angiogenesis and lymphangiogenesis in malignant tumors is accelerated by VEGF, and they may increase the frequency of blood and lymphatic metastases [31]. On the other hand, angiogenesis could also increase nutrient supply and contribute to tumor growth. In the present study, compared with PA, VEGF expression in RPA or CXPA tended to increase or increased significantly, and the increase in small microvessels in the tumor tissue was confirmed in the cross-sections from patients with CXPA by vWF immunostaining (Figure 4). Recently, it was suggested that there are structural and functional differences in the vasculature formed following neo-angiogenesis in malignant tumors. For example, it was reported that tumor vasculature is structurally and functionally aberrant, with intercellular junctions and extracellular matrix attachments that may not form normally in these tumors, leading to impaired monolayer formation and barrier function [32]. Moreover, because of the presence of endothelial abnormalities, the circulating immune cells cannot contact extra-capillary mutated malignant cells smoothly, and thus the process of tumor immune surveillance is decreased [33]. Unfortunately, we were unable to examine if there were structural differences in the vasculature among PA, RPA, and CXPA in the present study.

TGFβ1 is not only a key player in fibrosis in some organs, but it also acts as the TME and as a carcinomatous transformation regulator in cancer pathophysiology. For example, increased TGFβ1 levels not only suppress T cells’ anticancer function [34], but they also inhibit T-cell proliferation via decreasing the expression of interleukin-2 [35]; thus, the immune surveillance by T cells tends to decrease. Regarding the TME, TGFβ1 seemed to be important in cancer invasion and metastasis, as well as angiogenesis. For example, TGFβ1 plays an important role in cancer migration due to its mediation of CAF contractility and MMP secretion, where CAFs produce MMPs that destroy the structure of the TME architecture [36]. TGFβ1 was also reported to increase the expression of the angiogenic effectors VEGF, fibroblast growth factor (FGF), and MMPs, which regulate ECM degradation and remodeling to facilitate angiogenesis [37]. Interestingly, TGFβ1 is usually secreted in a latent form and can be activated by mast cell-derived chymase [14]. On the other hand, although MMP-9 activity was not measured in the present study, chymase was also reported to enzymatically cleave pro MMP-9 to the active form of MMP-9 [15]; thus, one can imagine the additive effects of TGFβ1 and MMP-9 generated by chymase on creating the TME in CXPA, and perhaps also on creating a special microenvironment for RPA. In the present study, the presence of TGFβ1 (Figure 9) and CAFs (Figure 2) in tumor tissues was immunohistologically confirmed, and it was also found that RPA was multi-nodular, and the tumor border in CXPA definitely became blurred (Figure 1), which may indicate that the ECM destruction by the active MMPs already happened in those tumor tissues from patients with CXPA and RPA.

Although accumulation of mast cells in solid tumors, such as gastric and lung cancers [16,17], has been reported in many clinical studies, few reports mentioned the reason why mast cells gather in these tumors. In the present study, it was found that glandular epithelial cells and cancer cells mainly expressed SCF in RPA and CXPA to some extent, and it is well known that SCF not only acts as a major chemotactic factor for mast cells and their progenitors, but also elicits cell–cell and cell–substratum adhesion, facilitates the proliferation, and sustains the survival, differentiation, and maturation of mast cells [38]. Surprisingly, it was reported that mast cell-derived chymase [10] could enzymatically cleave the membrane-bound SCF to release the bioactive form of SCF, indicating that an increase in the mast cells in RPA and CXPA may result from an increase in the chymase-containing MC_TC_-type cells.

As mentioned above, chymase is strongly associated with tumor growth and progress. Since the expression of MC_TC_ type cells tended to increase in RPA and CXPA compared with PA, the accumulation of mast cells containing chymase may give strong tumor activity even in parotid gland tumors. Confirmation of chymase expression in this study will lead to elucidation of the tumor activity of RPA and CXPA in future studies.

## 4. Limitations

This work focused only on the features of the histological distribution and the degree of chymase expression among three different types of parotid gland tumors. On the other hand, mast cells contain several mediators, and these may also be changed in RPA and CXPA. Therefore, one cannot reach a final conclusion until beneficial effects are obtained using a chymase-specific inhibitor in the animal models that resemble the pathophysiology of RPA and CXPA.

## 5. Materials and Methods

### 5.1. Sample Collection and Grouping

**Figure 10:**
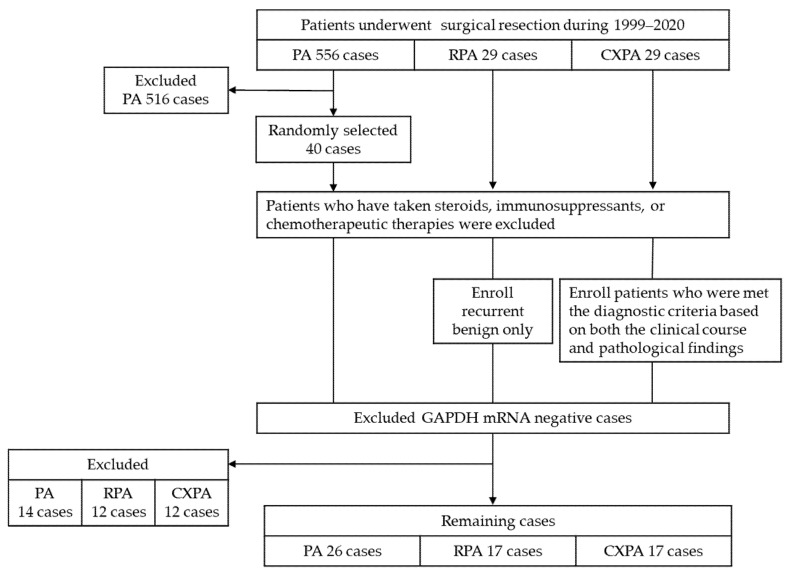
Schema of patient selection.

Surgical resections of parotid gland tumors performed during 1999–2020 in our hospital were included in this study. The resected tumor samples were fixed in 10% buffered formalin and embedded in paraffin until use in the present study. The tumors were diagnosed on the basis of the WHO classification [5]. During this time, a total of 566 cases underwent surgery for the treatment of PA, and 40 cases were chosen at random for the present study. There were 29 surgically resected samples enrolled in each of the RPA and CXPA groups. Patients who were found to have received steroids, immunosuppressants, or chemotherapeutic therapies were excluded from the present study. Criteria for enrollment in the RPA group were limited to benign recurrence only; tumor tissues with malignant lesions were excluded from present study. The cases enrolled in this group needed to meet the following criteria: there was no inconsistency with the diagnosis of CXPA based on both the clinical course and pathological findings. In addition, if the collected samples were negative for *GAPDH* mRNA expression, they were also excluded from the present study. Thus, 26 PA cases, 17 RPA cases, and 17 CXPA cases met the criteria (Figure 10). This study was performed in accordance with the ethical principles regarding human experimentation in the Declaration of Helsinki and approved by the Research Ethics Committee of Osaka Medical and Pharmaceutical University (authorization number: 2866-1).

### 5.2. General Histological and Immunohistological Studies

For histological and immunohistological staining, 4 μm-thick, serial cross-sections were prepared from paraffin blocks of PA, RPA, and CXPA using a microtome (LITORATOMU, REM-710, Yamato Koki Kogyo Ltd., Saitama, Japan).

The first serial cross-sections from each of the paraffin blocks were stained with hematoxylin and eosin (HE) to observe their general structures. On the seventh sections, Azan Mallory staining was performed to identify fibrosis. HE staining and Azan Mallory staining were performed in accordance with the standard staining protocols.

The third sections were stained with toluidine blue to identify mast cell distribution. In brief, deparaffinized sections were immersed in 0.5% toluidine blue solution (pH 4.8) for around 15 min, fractionated with 0.5% glacial acetic acid solution, and mounted after drying.

The second and fourth sections were used to show the distribution of chymase and tryptase using anti-chymase antibody (mouse monoclonal antibody against human mast cell chymase, 2D11G10D, 1:1000 dilution; a kind gift from Suzuki, Katakura Industries Co., Saitama, Japan) and anti-tryptase antibody (M7052, 1:800 dilution; Dako, Glostrup, Denmark), respectively.

The fifth and eleventh sections were used to stain for SCF (26582-1-AP, 1:200 dilution; Proteintech Group, Rosemont, IL, USA) and TGF-β1(ARP37894-P505, 1:100 dilution; AVIVA SYSTEMS BIOLOGY, San Diego, CA, USA). The sixth sections were used to stain for von Willebrand factor (vWF) (1:100 dilution; Dako, Glostrup, Denmark) to evaluate the degree of angiogenesis. To evaluate the mesenchymal cellular components (such as fibroblasts and myofibroblasts) among tumor tissues, vimentin (1:70 dilution; Cell Signaling Technology, Danvers, MA, USA) and α-smooth muscle actin (α-SMA) (1:200 dilution; Dako, Glostrup, Denmark) immunostainings were performed on the eighth and ninth sections. To identify the growth activity of the tumors, proliferating cell nuclear antigen (PCNA) (1:100 dilution; Dako, Glostrup, Denmark) immunostaining was also performed on the tenth sections.

Immunostaining with the abovementioned antibodies was performed in accordance with protocols described elsewhere [39,40]. In brief, deparaffinized sections were incubated with the respective antibodies overnight at 4 °C, followed by reaction with components from a labeled streptavidin-biotin peroxidase kit (Dako LSAB kit; Dako, Carpinteria, CA, USA). Thereafter, these sections were incubated with 3-amino-9-ethylcarbazole (AEC) for color development, counterstained with hematoxylin, and, finally, mounted with cover glasses.

The cellular number in each cross-section was counted in a high-power field (HPF:200×) in the five densest areas, and the average value of the five areas was used for statistical analysis.

### 5.3. Real-Time Reverse Transcriptase Polymerase Chain Reaction (RT-PCR)

Ten sheets of the 10 µm-thick paraffin sections were collected from the respective formalin-fixed, paraffin-embedded tissue blocks using a microtome to extract the RNA of the tissue, using methods described elsewhere [41]. Total RNA was extracted by the protocol provided in the total RNA isolation kit (ISOGEN PB Kit, NIPPON GENE Co., Ltd., Tokyo, Japan). Total RNA (2.5 µg) was transcribed into cDNA with SuperScript VILO (Invitrogen, Carlsbad, CA, USA). Then, mRNA levels were measured by RT-PCR on a Stratagene Mx3000P (Agilent Technologies, San Francisco, CA, USA) using Taq-Man fluorogenic probes. RT-PCR primers and probes for tryptase, chymase, vascular endothelial growth factor (VEGF), and glyceraldehyde-3-phosphate dehydrogenase (GAPDH) were designed by Roche Diagnostics (Tokyo, Japan). The primers were as follows: *5′-gatgctgagcctgctgct-3′* (forward) and *5′-gacgatacccgcttgctg-3′* (reverse) for *tryptase*, *5′-cattaacgggttcagttccag-3′* (forward) and *5′-agcaggaagggtcggttc-3′* (reverse) for *chymase*, *5′-gcagcttgagttaaacgaacg-3′* (forward) and *5′-ggttcccgaaaccctgag-3′* (reverse) for *VEGF*, and *5′-agccacatcgctcagacac-3′* (forward) and *5′-gcccaatacgaccaaatcc-3′* (reverse) for *GAPDH*. The probes were as follows: *5′-ctgcccca-3′* for *tryptase*, *5′-cagaggaa-3′* for *chymase*, *5′-ctccttcc-3′* for *VEGF*, and *5′-tggggaagg-3′* for *GAPDH*. The mRNA levels of *tryptase, chymase,* and *VEGF* were normalized to those of *GAPDH*.

### 5.4. Statistical Analysis

All numerical data are expressed as means ± SEM. Significant differences among the mean values of multiple groups were evaluated with one-way ANOVA followed by post hoc analysis (Fisher’s test). Fisher’s exact test and the Mann–Whitney U test were used to compare sex and age differences between respective groups. In all analyses, a *p*-value less than 0.05 was considered significant.

## 6. Conclusions

In the present study, in comparison with the benign tumor of PA, numbers of chymase-positive mast cells, as well as *chymase* gene expression, were markedly increased in RPA and CXPA. In view of the variety of effects of chymase on Ang I, latent TGFβ1, pro-MMPs, and membrane-binding SCF, the mast cell-derived chymase through its direct or cooperative effects with other mediators may participate in the pathophysiology of RPA and CXPA.

## Figures and Tables

**Table 1:** Demographic date of patients.

	PA		RPA		CXPA	
	Male	Female	Male	Female	Male	Female
Number	14	12	4	13	11	6
Age	56 (33–74)	58 (32–81)	42 (33–53)	58 (28–74)	64 (33–81)	61 (34–73)

## Data Availability

The data that support the findings of this study are available on request from the corresponding author, Denan Jin (E-mail: denan.jin@ompu.ac.jp).
